# Left Ventricle Size Correlates with Peak Exercise Capacity in Pediatric Cancer Survivors Exposed to Anthracycline Chemotherapy

**DOI:** 10.1007/s00246-023-03192-z

**Published:** 2023-05-22

**Authors:** Imran Ross Masood, Panteha Hayati Rezvan, Kyuwan Lee, Helena Vervaet, Christopher Kuo, Karla Loss, JonDavid Menteer, Andrew Souza, David Freyer, Jennifer A. Su

**Affiliations:** 1 Heart Institute, Children’s Hospital of Los Angeles, Los Angeles, USA; 2 Biostatistics and Data Analysis Core, The Saban Research Institute, Children’s Hospital Los Angeles, Los Angeles, USA; 3 Department of Population Sciences, City of Hope National Medical Center, Duarte, CA USA; 4 Present Address: Keck School of Medicine, University of Southern California, Los Angeles, USA; 5 Cancer and Blood Disease Institute, Children’s Hospital Los Angeles, Los Angeles, USA

**Keywords:** Anthracyclines, Exercise testing, Cancer survivor, Left ventricular function, Left ventricular size

## Abstract

Cancer survivors exposed to anthracycline chemotherapy are at risk for developing cardiomyopathy, which may have delayed clinical manifestation. In a retrospective cross-sectional study, we evaluated the utility of cardiopulmonary exercise testing (CPET) for detecting early cardiac disease in 35 pediatric cancer survivors by examining the associations between peak exercise capacity (measured via percent predicted peak VO_2_) and resting left ventricular (LV) function on echocardiography and cardiac magnetic resonance imaging (cMRI). We additionally assessed the relationships between LV size on resting echocardiography or cMRI and percent predicted peak VO_2_ since LV growth arrest can occur in anthracycline-exposed patients prior to changes in LV systolic function. We found reduced exercise capacity in this cohort, with low percent predicted peak VO_2_ (62%, IQR: 53–75%). While most patients in our pediatric cohort had normal LV systolic function, we observed associations between percent predicted peak VO_2_ and echocardiographic and cMRI measures of LV size. These findings indicate that CPET may be more sensitive in manifesting early anthracycline-induced cardiomyopathy than echocardiography in pediatric cancer survivors. Our study also highlights the importance of assessing LV size in addition to function in pediatric cancer survivors exposed to anthracyclines.

**Supplementary Information:**

The online version contains supplementary material available at 10.1007/s00246-023-03192-z.

## Introduction

Over the past few decades, treatment advances have significantly improved childhood cancer survival [1, 2]. Due to the intensive multimodal therapies, many survivors of childhood cancer develop clinically significant treatment-related toxicities, including anthracycline-induced cardiomyopathy [3–5]. Mulrooney et al. demonstrated an elevated risk of congestive heart failure associated with cumulative anthracycline treatment at doses of 250 mg/m^2^ or more among adult survivors of childhood and adolescent cancer [6]. Despite this association, early changes due to anthracycline-induced cardiomyopathy can be difficult to detect, and the rate of clinical progression is not well understood. It is known that a latent phase occurs between the initial anthracycline exposure and clinical manifestation of cardiomyopathy. Overt left ventricular (LV) dysfunction appears to be a later finding [7, 8]. Prior to cardiac dysfunction, LV growth arrest could be an early, subtle sign that manifests as reduced exercise capacity [9]. In a prospective study of LV size among adult cancer survivors exposed to anthracycline chemotherapy, LV mass declined over time and was associated with heart failure symptoms. This change in LV mass and functional limitation was shown to precede LV systolic dysfunction [10]. Therefore, attention should be given to early signs of LV pathology in pediatric patients exposed to anthracycline chemotherapy.

During the subclinical phase of anthracycline-mediated cardiomyopathy, LV growth arrest or LV dilation can occur [11]. Echocardiography and cardiac magnetic resonance imaging (cMRI) are essential tools for measuring LV size. While both tools are effective, they are limited in that measurements of myocardial performance are performed most commonly under resting conditions. Cardiopulmonary exercise testing (CPET), on the other hand, may detect subtle LV pathology that manifests during periods of physical activity prior to obvious changes in resting LV function seen by echocardiography or cMRI [12].

The primary objective of our study is to examine if peak exercise capacity, measured as percent predicted peak oxygen consumption (VO_2_) correlates with: (1) resting LV systolic function and (2) LV size, given that a growth-arrested LV could be an early and subtle indicator of anthracycline-induced cardiomyopathy. We hypothesized that variations in resting LV systolic function would not correlate with peak exercise capacity, and that LV size on resting echocardiography would be positively associated with percent predicted peak VO_2_. In anthracycline-exposed patients where LV growth arrest can occur, we secondarily analyzed the mechanisms of how LV size could impact exercise capacity. We examined if LV size correlated with the stroke volume response (percent predicted peak O2 pulse) and respiratory efficiency (Ve/VCO2) during exercise. We theorized that a smaller LV would result in a lower peak stroke volume. Since an elevated Ve/VCO2 slope can correlate with LV diastolic pressure, we hypothesized that a smaller LV would lead to a higher Ve/VCO2 slope.

## Materials and Methods

### Participants and Study Design

This was a retrospective cross-sectional study of patients who received anthracycline chemotherapy and underwent CPET at Children’s Hospital Los Angeles (CHLA), a quaternary-level facility. Inclusion criteria included patients who were referred to our cardiac anticipatory assessment and treatment (CAAT) Center for functional surveillance after anthracycline exposure. Referral to CAAT is typically made by the pediatric oncology team based on Children’s Oncology Group Long-term Follow-Up Guidelines for cardiac surveillance and had undergone CPET between 2018 and 2021 [13]. Patients in the CAAT clinic undergo further diagnostics such as cMRI or CPET per clinical surveillance protocol in those who received moderate or high dose anthracyclines. All patients had completed treatment and were in remission. All echocardiographic, cMRI, and CPET data were collected retrospectively in this study. For each patient, electronic medical record abstraction was used to record pertinent demographic and clinical data including age (in years), assigned sex at birth, race, body mass index (BMI (kg/m^2^)), cancer diagnosis, beginning and ending dates of cancer treatment, indexed cumulative anthracycline dose (mg/m^2^, converted to doxorubicin equivalent dose), thoracic radiation history, and dexrazoxane use. Patients without metabolic CPET data, those with submaximal exercise tests (determined as respiratory exchange ratio < 1.1, which are often excluded to improve the utility of exercise data), those on beta-block therapy, or those who had incomplete documentation of cumulative anthracycline dose were excluded. This study was approved as exempt by the CHLA institutional review board prior to collection of any study data.

### Cardiac Assessments

Transthoracic echocardiographic evaluation was performed using cardiac ultrasound imaging systems (Philips Medical Systems, Andover, MA) and was interpreted by board-certified pediatric cardiologists. LV function was defined by the ejection fraction (EF) and fractional shortening (FS). Resting LV function measured via EF was calculated in an apical 4-chamber view with bi-plane or Simpson’s measurements. An EF < 55% was considered abnormal. FS was calculated from a parasternal short axis view, and an abnormal FS was considered to be less than two Z-scores for BSA. If no Z-score was available, then an FS < 30% was considered abnormal. LV size assessment by echocardiography included the LV internal dimension in diastole (LVIDd), LV internal dimension in systole (LVIDs), and the LV mass (grams). Z-scores for echocardiographic parameters were normalized to body-surface area (BSA) and identified as normal if they were within 2 standard deviations (SDs) of the population mean. The echocardiogram performed most closely in time to the stress test was included. Stress echocardiograms were performed in a small subset of patients within the cohort along with CPET but were excluded to maintain uniformity.

Cardiac MRI was performed without sedation on a Philips Achieva at 1.5 T, using a cardiac or torso phased-array coil. Gadolinium contrast with gadopentetate dimeglumine (0.2 mm/kg) or gadobutrol (0.1 mm/kg) was hand injected at infusion rates of one to two cc per second. Cardiac mass and function were acquired using short axis steady state precession images covering both ventricles, and it was assessed using semiautomatic planimetry (with manual refinement). After approximately 10 min following contrast injection, delayed hyperenhancement imaging was performed using 2D inversion recovery gradient echo imaging with cardiac triggering at two R-R intervals. A 10-inversion time Look-Locker sequence was used to estimate the optimal inversion time to maximize myocardial nulling. This sequence was repeated, as needed, if myocardial nulling was inadequate on the delayed hyperenhancement imaging. All cMRI images were interpreted by board-certified pediatric cardiologists with advanced imaging training and between 5 and 22 years of dedicated cMRI experience. LV function by cMRI was assessed by the LVEF. The LV volume by cMRI was primarily defined as the LV end-diastolic volume (LVEDV). Another marker of LV size by cMRI was the LV mass, which is a summative calculation of the LV mass in grams. Z-scores of the LVEDV and LV mass were also measured in order to account for discrepancies in patient BSA. Cardiac index (CI) was calculated using cardiac output by flow measurement, divided by the patient’s BSA. Assessment of myocardial fibrosis by cMRI was performed by measurement of late gadolinium enhancement (LGE) and by percentage of extracellular volume (ECV). An abnormal ECV was defined as being > 30% of myocardial fluid volume [14].

Exercise testing was performed using a standard ramp protocol on cycle ergometry. No treadmill tests were included due to a lack of metabolic data. All tests were supervised by a pediatric cardiologist. Metabolic data were collected using a Carefusion Vsense encore metabolic cart (Carefusion, Yorba Linda, CA) and normative values were determined using the Cooper equation [15]. We defined peak exercise capacity as the percent predicted peak VO_2_ during CPET. An abnormal percent predicted peak VO_2_ was considered < 80%. Chronotropic incompetence was defined as a peak heart rate (HR) less than 80% of expected peak HR for age (220—age in years). The expected peak work rate is considered 3–3.5 W/kg. An abnormal Ve/VCO_2_ (ratio of minute ventilation to volume of exhaled CO_2_, representative of lung ventilation-perfusion (V/Q) matching) slope was defined as 28 or above. A normal breathing reserve was defined as 20–40% of maximal ventilation. Peak O_2_ pulse was defined as the (VO_2_ peak)/(peak HR), which serves a surrogate for LV stroke volume.

### Statistical Analysis

Continuous data are presented as median and interquartile range [IQR] and categorical data as frequency and percentage. The Mann–Whitney U test and Kruskal–Wallis test were used to determine whether percent predicted peak VO_2_ differed by sex and race, respectively. Correlations of LV size measures were calculated using Pearson correlation (*r*). As part of univariate analyses, we used linear regression models to examine individual associations between percent predicted peak VO_2_ and demographic and clinical characteristics, along with echocardiographic measurements of LV function (i.e., EF and FS) and LV size (i.e., LVIDd, LVIDd Z-score, LVIDs, LVIDs Z-score, LV mass, and LV mass Z-score). We then used a multiple linear regression model, including factors that were expected to be important in predicting the outcome for substantive reasons such as age, sex, and BMI, along with factors that were significantly associated with the primary outcome of interest in the univariate analyses. To avoid unstable estimates, we performed diagnostic tests for multicollinearity levels. When collinearity was present, we removed the factors that were highly correlated from the multivariable model and constructed multivariable models to determine the relationships of these factors with percent predicted peak VO_2_ separately. Using the same strategy described above, we further examined univariate and multivariable associations between echocardiographic parameters of LV size and the secondary outcomes—percent predicted peak O_2_ pulse and Ve/VCO_2_ slope.

The primary outcome, percent predicted peak VO_2_, was a fully observed variable, and the secondary outcomes had 2.9% missing data. While the echocardiography data were available on all the eligible patients, cMRI data were only observed in 60% of them, where LV function and size measurements (LVEF, LVEDV Z-score, LV mass, and LV mass Z-score) had more than 40% missing values. Due to high percentage of missing cMRI data and to avoid heterogeneity of imaging modalities, we did not proceed with performing multivariable analyses using the cMRI data.

All statistical analyses were performed using Stata/MP version-17.0 [16]. Two-tailed statistical tests for the parameter estimates were conducted with α = 0.05, where an a priori level of significance was considered at less than 0.05.

## Results

### Demographic and Clinical Data

Among 817 exercise tests performed during the study period, 36 patients had received anthracycline chemotherapy and met inclusion criteria. Of these, 35 had technically satisfactory outcomes data and formed the analytic cohort. One patient was excluded for having achieved a peak respiratory exchange ratio < 1.1. Demographic and clinical data of the study cohort are outlined in Table 1. The median age was 17 years (IQR 14–19); almost half of the patients (51.4%) assigned male at birth; 57.1% were Latinx and 22.9% were White. The median BMI was 23.8 kg/m^2^ (IQR 18.7–26.9). The most common oncologic diagnoses were acute lymphocytic leukemia (ALL, 29%) and Hodgkin lymphoma (20%). Median time from initial anthracycline exposure was close to 7 years (IQR 4.8–14) and the median cumulative doxorubicin dose was 300 mg/m^2^ (IQR 250–450). Dexrazoxane was used in 20% of patients (n = 7); and 20% had an unknown history of dexrazoxane use. Thoracic radiation was utilized in 31.4% of patients (n = 11).

**Table 1:** Demographic and clinical data (n = 35)

Patient Characteristics	n	(%)	
Age (years), median [IQR]	17		[14–19]
Sex (Male)	18		(51.4%)
Race			
Latinx	20		(57.1%)
White	8		(22.9%)
Asian	2		(5.7%)
Other	5		(14.3%)
BMI (kg/m^2^), median [IQR]	23.8		[18.7–26.9]
Oncologic diagnosis			
ALL	10		(29%)
Hodgkin’s lymphoma	7		(20%)
AML	6		(17%)
Osteosarcoma	3		(9%)
Other^¥^	9		(26%)
Time since anthracycline exposure (years), median [IQR]	6.6		[4.8–14]
Cumulative doxorubicin dose (mg/m^2^)^†^, median [IQR]	300		[250–450]
Cumulative doxorubicin dose > 250 mg/m^2^	25		75.8%
Dexrazoxane use	7		25%
Thoracic radiation exposure	11		31.4%

Data are frequency and percentage% or median [IQR]

*ALL* acute lymphocytic leukemia, *AML* acute myelocytic leukemia

^¥^Other diagnoses include Ewing’s sarcoma [3], Wilm’s tumor [2], and other [3]

^†^The frequency and percentage of incomplete variables: Time since anthracycline exposure (n = 8, 22.9%); Doxorubicin dose (n = 2, 5.7%); Dexrazoxane use (n = 7, 20%)

### Echocardiography

Routine clinical echocardiograms were performed on all 35 patients in this cohort, and all were done within 8 months of CPET (Table 2). LV function (measured as EF or FS) was normal in the majority of the cohort (85.7%, n = 30). The EF was measured in 30 patients, with a median of 59% (IQR 57–62%). FS was measured in 35 patients with a median of 33.3% (IQR 30.6–36.4%). The LVIDd Z-score was normal in almost all the patients (94%, n = 33) with median − 0.5 (IQR − 1.5 to 0.2). The median LVIDs Z-score was − 0.1 [− 0.8 to 0.6]. Median LV mass Z-score by echocardiography was low, with a median of − 1.6 [− 2.1 to − 0.8]. In the two patients with a low LVIDd Z-score (< − 2 SDs), LV mass Z-score was also noted to be low (< − 2 SDs). No other significant cardiac anatomic anomalies were observed.

**Table 2:** Echocardiography and cMRI results

Echocardiography (n = 35)	Median [IQR]	Mean (SD)
LV function		
EF (%)^†^	59 [57–62]	59% (0.1)
FS (%)	33.3 [30.6–36.4]	32.4 (7.6)
LV size		
LVIDd (cm)	4.5 [4.2–5.2]	4.7 (0.5)
LVIDd Z-score	− 0.5 [− 1.5 to 0.2]	− 0.6 (1.2)
LVIDs (cm)	3 [2.7–3.5]	3.1 (0.5)
LVIDs Z-score^†^	− 0.1 [− 0.8 to 0.6]	− 0.01 (1.2)
LV mass (g)	110.2 [88.5–141.6]	117 (37.1)
LV mass Z-score^†^	− 1.6 [− 2.1 to − 0.8]	− 1.4 (1)
LV mass to LVIDd ratio	24.6 [19.4–30]	24.7 (5.9)
cMRI (n = 21)		
LV function		
EF (%)^†^	57.8 [55.8–64.1]	59.2% (7.1)
CI (L/min/m^2^)^†^	3.0 [2.7–3.5]	3.1 (0.5)
LV size		
LVEDV (mL)^†^	104.1 [80.5–150.5]	116.7 (45.9)
LVEDV Z-score^†^	− 2.0 [− 3.4 to − 1.1]	− 2.2 (1.6)
LV mass (g)^†^	86.8 [63.5–109.7]	88.7 (28.4)
LV mass Z-score^†^	− 2.2 [− 2.6 to − 1.4]	− 1.9 (1.6)
LVEDV to LV mass ratio^†^	1.4 [1.1–1.7]	1.4 (0.4)

*EF* ejection fraction, *FS* fractional shortening, *LVIDd* LV internal dimension in diastole, *LVIDs* LV internal dimension in systole, *CI* cardiac index, *LVEDV* LV end-diastolic volume

^†^The frequency and percentage of incomplete variables: EF by echo (n = 5, 14.3%); LVIDs Z-score (n = 1, 2.9%); LV mass Z-score by echo (n = 1, 2.9%); EF by cMRI (n = 14, 40%); CI (n = 21, 60%); LVEDV (n = 14, 40%); LVEDV Z-score (n = 15, 42.9%); LV mass by cMRI (n = 19, 54.3%); LV mass Z-score by cMRI (n = 19, 54.3%); LVEDV to LV mass ratio (n = 19, 54.3%)

### Cardiac Magnetic Resonance Imaging

Cardiac MRI was performed in 60% of the patients in this cohort (n = 21). Table 2 presents full cMRI data. An LVEDV was measured in 21 patients; half of the patients had a normal LVEDV (median: 104.1, IQR 80.5–150.5) and the other half had decreased LVEDV. Myocardial fibrosis was not identified in any patient. The LV mass was measured in 16 patients, where the median was 86.8 g (IQR 63.5–109.7) and the median Z-score was − 2.2 (IQR − 2.6 to − 1.4). CI was measured in 14 patients of the 21 patients with cMRI data, with a median of 3.0 L/min/m^2^ (IQR 2.7–3.5).

### Cardiopulmonary Exercise Testing

Full exercise data is shown in Table 3. Exercise capacity was reduced in this cohort, with low median peak work rate of 2.2 W/kg (IQR 1.7–2.7). The peak VO_2_ and percent predicted peak VO_2_ were also low, with a median of 25.3 mL/kg/min (IQR 21.9–33.9) and 62% (IQR 53–75), respectively. The majority of patients (86%, n = 30) had a peak VO_2_ below 80% of predicted. Additionally, the median percent predicted O_2_ pulse was 81.5% (IQR 73–99). Ve/VCO_2_ slope was measured in 34 patients. The median Ve/VCO_2_ slope was 28 (IQR 27–31) and was abnormally elevated in 21 patients (62%). The breathing reserve was normal in all patients.

**Table 3:** Exercise data (n = 35), including resting and peak findings

	Median [IQR]	Mean (SD)
Peak work rate (W/kg)	2.2 [1.7–2.7]	2.2 (0.7)
RER	1.2 [1.2–1.3]	1.3 (0.1)
Peak VO_2_ (L/min)	1.7 [1.2–2.2]	1.7 (0.6)
Adjusted VO_2_ (mL/kg/min)	25.3 [21.9–33.9]	27.2 (6.9)
Percent predicted Peak VO_2_	62 [53–75]	63.4 (13.7)
VO_2_ at AT (mL/kg/min)	16.5 [12.6–18.7]	16.4 (4.3)
O_2_ pulse (mL/beat)	8.9 [6.6–12.3]	9.6 (3.4)
Percent predicted peak O_2_ pulse	81.5 [73–99]	83.1 (16.3)
Ve/VCO_2_ slope	28 [27–31]	28.8 (3.3)
Resting HR (bpm)	91 [82–101]	91.6 (15.6)
Peak HR (bpm)	187 [176–193]	185 (11.2)
Resting sBP (mmHg)	113.5 [105–120]	111.9 (12.5)
Peak sBP (mmHg)	155.5 [135.3–178]	157.9 (30.8)
Resting dBP (mmHg)	70 [62–77]	69.4 (8.4)
Peak dBP (mmHg)	70 [60–80]	71.4 (14.7)
Baseline MAP (mmHg)	72.9 [64.2–78.5]	72 (7.6)
Peak MAP (mmHg)	83.3 [79.5–96.3]	88.3 (15.2)

Data are frequency and percentage % or median [IQR] and mean (SD)

*RER* respiratory exchange ratio, *VO*_*2*_ oxygen consumption, *AT* anaerobic threshold, *Ve/VCO*_*2*_ ventilatory equivalents/CO_2_ production, *HR* heart rate, *Bpm* beats per minute, *sBP* systolic blood pressure, *dBP* diastolic blood pressure, *MAP* mean arterial pressure

^†^The frequency and percentage of incomplete variables: Peak work rate (n = 1, 2.8%), VO_2_ at AT (n = 11, 31%), O_2_ pulse and percent predicted O_2_ pulse (n = 1, 2.8%), Ve/VCO_2_ slope (n = 1, 2.8%)

### Factors Associated with Exercise Capacity

The results of univariate analyses of associations between percent predicted peak VO_2_ and demographics, clinical characteristics, and echocardiographic parameters are shown in Table 4. There were no meaningful differences in distributions of percent predicted peak VO_2_ observed by sex or race. The estimated mean change in percent predicted peak VO_2_ with each one-unit (kg/m^2^) increase in BMI was − 0.97 (95% CI − 1.67, − 0.28, p = 0.008). We did not find sufficient evidence indicating relationships between percent predicted peak VO_2_ and oncologic diagnosis, time from doxorubicin exposure, cumulative anthracycline dose, thoracic radiation exposure, and use of dexrazoxane (Table 4).

**Table 4:** Estimated regression coefficient with 95% confidence interval (CI) obtained from a univariate analysis for the analysis of the association between percent predicted peak VO_2_ and demographic and clinical characteristics, echocardiogram, and cMRI measures

	Estimate	95% CI
Demographic and clinical characteristics		
Age (years)	− 0.33	(− 1.87, 1.21)
Sex (Male)	− 6.95	(− 2.27, 16.17)
Race		
Latinx	− 5.65	(− 18.06, 6.76)
White	− 6	(− 20.63, 8.63)
Asian/Other		
BMI (kg/m^2^)^*^	− 0.97	(− 1.67, − 0.28)
Oncologic diagnosis		
ALL/AML	7.95	(− 3.51, 19.41)
Hodgkin’s Lymphoma	0.46	(− 13.40, 14.31)
Osteosarcoma	− 5.11	(− 23.44, 13.22)
Other^¥^		
Time since anthracycline exposure (years)^†^	− 0.04	(− 1.08, 1.00)
Cumulative doxorubicin dose (mg/m^2^)	− 0.01	(− 0.05, 0.03)
Cumulative doxorubicin dose > 250 mg/m^2^	− 3.04	(− 14.57, − 8.49)
Dexrazoxane use	− 8.81	(− 20.57, 2.96)
Thoracic radiation exposure	− 7.6	(− 17.52, 2.31)
Echocardiography		
LV function		
EF (%)^†^	21.04	(− 78.22, 120.30)
FS (%)	− 0.48	(− 1.09, 0.13)
LV size		
LVIDd (cm)	5.47	(− 3.15, 14.10)
LVIDd Z-score*	5.14	(1.52, 8.76)
LVIDs (cm)	5.64	(− 4.17, 15.44)
LVIDs Z-score*	4.32	(0.58, 8.06)
LV Mass (g)	0.02	(− 0.11, 0.15)
LV Mass Z-score*^†^	7.57	(3.61, 11.54)
LV Mass/LVIDd ratio	− 0.05	(− 0.86, 0.77)
cMRI		
LV function		
EF (%)^†^	0.14	(− 0.69, 0.97)
CI (L/min/m^2^)^†^	3.11	(− 9.54, 15.76)
LV size		
LVEDV (mL)^†^	0.003	(− 0.12, 0.13)
LVEDV Z-score*^†^	4.17	(1.15, 7.19)
LV mass (g)^†^	0.12	(− 0.10, 0.34)
LV mass Z-score^†^	2.32	(− 1.70, 6.34)
LVEDV to LV mass ratio	11.54	(− 5.04, 28.13)

*ALL* acute lymphocytic leukemia, *AML* acute myelocytic leukemia, *EF* ejection fraction, *FS* fractional shortening, *LVIDd* LV internal dimension in diastole, *LVIDs* LV internal dimension in systole, *LVEDV* LV end-diastolic volume

*p-value < 0.05

^¥^Other diagnoses include Ewing’s sarcoma [3], Wilm’s tumor [2] and other [3]

^†^The frequency and percentage of incomplete variables: Time since anthracycline exposure (n = 8, 22.9%); Doxorubicin dose (n = 2, 5.7%), Dexrazoxane use (n = 7, 20%); EF by echo (n = 5, 14.3%); LVIDs Z-score by echo (n = 1, 2.9%); LV mass z-score by echo (n = 1, 2.9%); EF by cMRI (n = 14, 40%); CI (n = 7, 33%); LVEDV (n = 14, 40%); LVEDV Z-score (n = 15, 42.9%); LV mass by cMRI (n = 19, 54.3%); LV mass Z-score (n = 19, 54.3%); LVEDV to LV mass ratio (n = 19, 54.3%)

There was a positive association between percent predicted peak VO_2_ and LV size by echocardiography (Table 4). In particular, the estimated mean change in percent predicted VO_2_ associated with one-unit of increase in: (1) LVIDd Z-score was 5.14 (95% CI 1.52, 8.76, p = 0.007), (2) LVIDs Z-score was 4.32 (95% CI 0.58, 8.06, p = 0.025), and (3) LV mass Z-score was 7.57 (95% CI 3.61, 11.54, p < 0.001). We did not find evidence indicating associations between percent predicted peak VO_2_ and LV size by cMRI, except with LVEDV Z-score, where for every increase in LVEDV Z-score percent predicted peak VO_2_, on average, increased by 4.17 (95% CI 1.15, 7.19, p = 0.009). Eighty-five percent of this pediatric cohort had quantitatively normal systolic LV function as measured by EF and FS. No significant associations were observed between percent predicted peak VO_2_ and echocardiographic and cMRI measurements of resting LV function (Table 4).

Table 5 summarizes the findings from multivariable linear regression models of associations of LVIDd Z-score, LVIDs Z-score, and LV mass Z-score with percent predicted peak VO_2_. Given the strong correlation between LVIDd Z-score and LVIDs z-score (*r* = 0.79), moderate correlations between LVIDd Z-score and LV mass (*r* = 0.58), and LVIDs Z-score and LV mass (*r* = 0.51), and the existence of multicollinearity, we constructed three separate models with LVIDd Z-score, LVIDs z-score, and LV mass Z-score, adjusting for age, sex, and BMI. An additional multivariable linear regression model was formed with both LVIDd Z-score and LV mass Z-score including age, sex, and BMI (Model 4), where LVIDs Z-score was excluded due to fairly strong positive correlation with the LVIDd Z-score and its lower-yield clinical utility.

**Table 5:** Estimated regression coefficient with 95% confidence interval (CI) obtained from multivariable regression models for the analysis of the association between percent predicted VO_2_ and echocardiographic measures of LV size

	Model 1		Model 2		Model 3		Model 4	
	Estimate	95% CI	Estimate	95% CI	Estimate	95% CI	Estimate	95% CI
Age (years)	− 0.38	(− 1.70, 0.93)	− 0.49	(− 1.83, 0.85)	− 0.32	(− 1.46, 0.82)	− 0.34	(− 1.48, 0.81)
Sex (Male)	8.90*	(0.58, 17.21)	9.48*	(1.54, 17.41)	6.20	(0.80, 13.20)	5.79	(− 1.30, 12.88)
BMI (kg/m2)	− 0.97*	(− 1.62, − 0.31)	− 1.21*	(− 1.86, − 0.55)	− 1.25*	(− 1.80, − 0.69)	− 1.21*	(− 1.78, − 0.65)
LVIDd Z-score	3.42	(− 0.03, 6.87)					1.67	(− 2.05, 5.38)
LVIDs Z-score			3.99*	(0.81, 7.17)				
LV Mass Z-score					7.32*	(4.00, 10.65)	6.41*	(2.50, 10.32)

*_p-value < 0.05_

According to Table 5 (Models 1–3), the relationship between LV size and peak exercise capacity remained significant after accounting for age, sex, and BMI. In particular, when comparing patients with identical age, sex, and BMI, the estimated mean change in percent predicted peak VO_2_ was: 3.99 (95% CI 0.81, 7.17, p = 0.016) for every unit increase in LVIDs Z-score and 7.32 (95% CI 4.00, 10.65, p < 0.001) for every unit increase in LV mass Z-score. LVIDd Z-score showed a borderline significant association, where for its every unit increase, the estimated change in average percent predicted peak VO_2_ was 3.42 (95% CI − 0.03, 6.87, p = 0.052). As shown in Model 4, the association between LV mass Z-score and percent predicted peak VO_2_ was somewhat attenuated but remained significant, where the average rate of change in percent predicted peak VO_2_ was 6.41 (95% CI 2.50, 10.32, p = 0.002) for patients of the same age, sex, BMI, and LVIDd Z-score but one-unit different in LV mass Z-score. The association between percent predicted peak VO_2_ and LVIDd Z-score was considerably weakened (1.67, 95% CI − 2.05, 5.38, p = 0.366) and became non-significant with all the other covariates held constant. We further explored the interaction effects of age, sex, and BMI with LV function and LV size in separate models for analyses of percent predicted peak VO_2_ and did not find evidence of significant interaction.

Additional univariate analyses (Supplementary Table 1) show that the estimated change in average percent predicted peak O_2_ pulse per difference of one-unit (kg/m^2^) in BMI was 1.08 (95% CI 0.23, 1.93, p = 0.015), the estimated difference in average Ve/VCO_2_ slope between males and females was − 4.35 (95% CI − 6.07, − 2.62, p < 0.001), and the estimated difference in average Ve/VCO_2_ slope for patients differing by one-unit (kg/m^2^) of BMI was − 0.22 (95% CI − 0.40, − 0.03, p = 0.022).

We found evidence of associations between LV size measurements and percent predicted peak O_2_ pulse and Ve/VCO_2_ slope (Supplementary Table 1). In particular, the expected difference in predicted peak O_2_ pulse for every one-unit difference in: (1) LVIDd Z-score was 6.01 (95% CI 1.61, 10.41, p = 0.009), (2) LVIDs Z-score was 5.43 (95% CI 1.16, 9.71, p = 0.014), and (3) LV mass Z-score was 8.1 (95% CI 3.34, 12.86, p = 0.002). While no evidence of significant associations was observed between Ve/VCO_2_ slope and LVIDd Z-score and LVIDs Z-score, the estimated mean change in Ve/VCO_2_ slope was − 1.16 (95% CI − 2.22, − 0.11, p = 0.032) for every increase in LV mass Z-score, suggesting that a higher LV mass Z-score was associated with a more efficient ventilation/perfusion balance during exercise.

Comparing patients with the same sex, age, and BMI, the estimated change in mean percent predicted peak O_2_ pulse was 7.44 (95% CI 3.57, 11.32, p < 0.001) for every one-unit difference in LVIDd Z-score, 5.13 (95% CI 1.39, 8.86, p = 0.009) for every one-unit difference in LVIDs Z-score, and 7.83 (95% CI 3.55, 12.10, p = 0.001) for every one-unit difference in LV mass Z-score (Supplementary Table 2: Models 1–3). According to model 4, the average rate of change in percent predicted peak O_2_ pulse decreased to 5.86 (95% CI 1.03, 10.70, p = 0.019) for patients with the same age, sex, and BMI who differ by one-unit on LV mass Z-score. The association between predicted peak O_2_ pulse and LVIDd Z-score was not significant (3.75, 95% CI − 0.97, 8.47, p = 0.115), while holding all other covariates constant. The association between LV mass Z-score and Ve/VCO_2_ slope was no longer significant after taking into account age, sex, and BMI in multivariable models (Supplementary Table 3: Models 3–4).

## Discussion

Cancer survivors exposed to anthracycline chemotherapy are at risk for developing cardiomyopathy. In pediatric patients, cardiac manifestations of anthracycline toxicity can be delayed. It is hypothesized that anthracycline therapy and radiation impair cardiac myocyte growth, leading to reduced ventricular volume and cardiac reserve, and/or wall thinning and increased wall stress [10]. Our study evaluated the utility of CPET for detecting functional limitations in pediatric cancer survivors and compared these findings to routine echocardiography and cMRI. Indeed, we found consistently reduced exercise capacity in this cohort, even as the majority of patients in our cohort maintained quantitively normal LV systolic function. Importantly, we observed an idiosyncratic reduction in LV size in this study cohort, and significant associations between LV size and peak exercise capacity and peak stroke volume achieved during exercise.

Previous studies have reported similarly diminished exercise capacity in anthracycline recipients [17–19]. The breathing reserve was normal in all patients, indicating that exercise performance was not limited by respiratory mechanics. The mechanism of reduced exercise capacity is likely multifactorial. In addition to anthracycline cardiotoxicity, other contributing factors include deconditioning, skeletal muscle weakness, and activity limitation due to chronic illness. For example, sarcopenia is associated with anthracycline-cardiomyopathy [20], and it is known that skeletal muscle mass is a predictor of peak VO_2_ [21, 22].

Anthracycline cardiomyopathy in our cohort was subtle by imaging assessment, with normal systolic LV function in 85.7% of patients by echocardiography despite the majority of patients having an abnormally low percent predicted peak VO_2_. This suggests that children exposed to anthracyclines may have normal systolic function at rest, however, are unable to regulate the various mechanisms that are required to sufficiently augment cardiac output on demand, reflecting poor cardiac reserve. Similarly, 94% of our cohort demonstrated a quantitively normal LV size by echocardiogram. Despite this, we found a notable relationship between LV size and peak exercise capacity. In particular, we found univariate connections between LVIDd Z-score, LVEDV Z-score by cMRI, and percent predicted peak VO_2_. The LV mass Z-score by echocardiography was also positively associated with percent predicted peak VO_2_. This association remained significant after further inclusion of sex, age, and BMI in the multivariable regression model. Cardiac MRI did not reveal fibrosis in our patients with small LV cavities. The authors therefore theorize that in the context of growth-arrest due to anthracycline exposure, a smaller LV predisposes to a lower stroke volume, which may impair peak exercise performance. The exact pathophysiologic mechanisms for association between LV size and peak exercise capacity in anthracycline recipients requires further exploration.

In our cohort, we also observed a lower stroke volume response to peak exercise as assessed by percent predicted peak O_2_ (Table 3). This stroke volume response to peak exercise correlated with LV cavity size (see Supplemental Materials). A similar submaximal stroke volume response has been previously demonstrated, when assessing ΔHR/Δ work rate as a surrogate for stroke volume [23]. During peak exercise, a normal individual can be expected to augment stroke volume by up to 25%. Anthracycline recipients with a smaller LV cavity will likely have lower reserve to increase LV preload, resulting in suboptimal stroke volume at peak exercise and adversely impacting peak exercise performance. Additionally, an individual’s level of physical conditioning is known to affect baseline and peak stroke volume [24]. Likely, the diminished LV cavity and low stroke observed in our cohort is multifactorial, a result both of relative deconditioning from chronic disease in addition to anthracycline cardiotoxicity.

Previous data has demonstrated that LV diastolic dysfunction can occur after anthracycline chemotherapy, often measured non-invasively by abnormal mitral annulus tissue Doppler velocity [18, 25]. As heart rates increase with exercise, rapid LV relaxation plays an important role in maintaining stroke volume and cardiac output as diastolic filling time decreases. Our study evaluated diastolic dysfunction by using Ve/VCO_2_ slope as a reasonable surrogate, as Ve/VCO_2_ slope has been shown to correlate with pulmonary artery pressures and pulmonary vascular resistance [25]. We did not find significant associations between Ve/VCO_2_ slope and LV cavitary size. There was, however, a weak negative association between LV mass Z-score by echocardiography and the Ve/VCO_2_ slope, suggesting that diminished LV mass portends an unfavorable ventilation-perfusion mismatch during exercise.

In 2014 Lipshultz et al. described a phenomenon of LV growth arrest amongst patients who received anthracyclines, an entity known as “Grinch syndrome” [26]. Following growth arrest, patient develop one of several phenotypes. Either the heart can grow in volume with inadequate myocardium, leading to thin-walled, high-wall stress physiology; or the heart can fail to gain volume and maintain normal thickness and wall stress at the expense of stroke volume. Diastolic dysfunction is prominent in this population [27–29] and severely affected patients may develop long-term complications similar to those of primary restrictive cardiomyopathy. In our cohort, we also observed the average LV mass Z-score to be low, but both the normal volume and reduced volume phenotypes were seen. Further studies to evaluate the associations between exercise conditioning on LV size and exercise capacity may help to inform whether our observation of mildly decreased LV dimension is primarily a result of cardiovascular deconditioning, or perhaps, an early sign of myocardial growth restriction from anthracycline exposure. Likely, both are contributing factors. Further evaluation of why anthracycline-recipients are at risk for having smaller LV size and resultant lower exercise capacity, as well as determination whether of these findings are modifiable with lifestyle intervention will have tremendous implications in terms of management goals and counseling for these patients.

With regard to cardiac screening for anthracycline-recipients, this study suggests that routine resting echocardiography may provide only a partial picture of the cardiovascular health in anthracycline recipients. CPET appears to be more sensitive in this population, allowing assessment of cardiopulmonary performance during active state, and thus, provides valuable information in the evaluation of early anthracycline-cardiomyopathy beyond what can be assessed for by resting echocardiogram alone. Furthermore, based on findings from our study, abnormalities on CPET appear to precede functional impairment by resting echocardiography or cMRI along the course of anthracycline-mediated cardiomyopathy disease progression.

## Strengths and Weaknesses

This study revealed that children exposed to anthracycline chemotherapy are at risk of reduced exercise capacity, despite having normal resting LV function. We highlighted that LV cavitary size is reduced among anthracycline-recipients, and also correlates to exercise capacity. For children exposed to anthracycline chemotherapy, manifestations of cardiomyopathy can take years to manifest, and this study revealed the utility of performing CPET prior to overt cardiomyopathy develops.

Our study was limited by its sample size, retrospective analysis, lack of control group, and cross-sectional design, which cannot establish causality. cMRI data was incomplete and therefore, limited our analyses and generalizability of the findings. A large majority of exercise data from this study was measured during the COVID-19 pandemic, a time of quarantine and physical deconditioning, and therefore, could potentially underestimated true exercise capacity among anthracycline-recipients [30]. Future studies could explore the relationships of LV size measures and exercise capacity using longitudinal data. Understanding exercise capacity may also open future avenues for targeted pediatric rehabilitation programs for children exposed to anthracyclines [31].

## Conclusion

In conclusion, decreased exercise capacity is common in children following treatment with anthracyclines, and is present despite normal resting systolic function measured by routine echocardiography. Rather, decreased LV size as described by LVIDd and LV mass by echocardiography and LVEDV using cMRI, appear to be associated with exercise capacity. This study highlights the utility of measuring CPET as well the importance of LV size measurements by echocardiography and cMRI. Decreased LV dimensions are likely due LV growth arrest following anthracycline exposure. The inability for a diminutive LV to appropriately augment stroke volume may be a limitation to peak exercise. It is possible that an early manifestation of cardiac disease among anthracycline-recipients is reduced exercise capacity, a reflection of reduced cardiac reserve, and in part related to LV size. Providers who care for anthracycline-recipients should consider referral for CPET as part of a cardiovascular health evaluation and to detect early stages of anthracycline cardiomyopathy.

## Supplementary Information

Below is the link to the electronic supplementary material.

Supplementary file1 (DOCX 20 kb)
