# Supplementary material for: Left Ventricle Size Correlates with Peak Exercise Capacity in Pediatric Cancer Survivors Exposed to Anthracycline Chemotherapy
Source: Pediatr Cardiol. 2023 May 22;45(7):1493–502. doi: 10.1007/s00246-023-03192-z (PMC10202348; doi:10.1007/s00246-023-03192-z)
Supplement: Supplementary file 1 — Supplementary file1 (DOCX 20 kb) [file 246_2023_3192_MOESM1_ESM.docx]

# Supplementary Materials

| **Table S1**. Estimated regression coefficient with 95% confidence interval (CI) obtained from a univariate analysis for the analysis of the association between 1) percent predicted peak O_2_ pulse and 2) Ve/VCO_2_ slope and demographic and clinical characteristics | | | | | | |
| --- | --- | --- | --- | --- | --- | --- |
|  | | **Percent predicted O_2_ pulse** | |  | **Ve/VCO_2_ slope** | |
|  | | **Estimate** | **95% CI** |  | **Estimate** | **95% CI** |
| **Demographic and clinical characteristics** | |  |  |  |  |  |
| Age (years) | | -1.2 | (-3.01, 0.59) |  | -0.25 | (-0.61, 0.11) |
| Sex (Male) | | 0.47 | (-11.06, 12.0) |  | -4.35^*^ | (-6.07, -2.62) |
| Race | |  |  |  |  |  |
|  | Latinx | 0.6 | (-6.7, 7.9) |  | -0.54 | (-2.0, 0.9) |
|  | White | -4.6 | (-13.5, 4.2) |  | 1.7 | (-0.1, 3.4) |
|  | Asian/Other |  |  |  |  |  |
| BMI (kg/m^2^) | | 1.08^*^ | (0.23, 1.93) |  | -0.22^*^ | (-0.4, -0.03) |
|  | |  |  |  |  |  |
| **Echocardiography**  LVIDd Z-score | | 6.01^*^ | (1.61, 10.41) |  | -0.24 | (-1.21, 0.73) |
| LVIDs Z-score | | 5.43^*^ | (1.16, 9.71) |  | -0.26 | (-1.21, 0.69) |
| LV Mass Z-score | | 8.10^*^ | (3.34, 12.86) |  | -1.16^*^ | (-2.22, -0.11) |
|  | |  |  |  |  |  |
| **cMRI** | |  |  |  |  |  |
| LVEDV Z-score | | 4.92 | (-0.02, 9.86) |  | 0.60 | (-0.33, 1.53) |

^*^p-value <0.05

| **Table S2.** Estimated regression coefficient with 95% confidence interval (CI) obtained from multivariable regression models for the analysis of the association between percent predicted O_2_ pulse and echocardiographic measures of LV size | | | | | | | | | | | |
| --- | --- | --- | --- | --- | --- | --- | --- | --- | --- | --- | --- |
|  | **Model 1** | | | **Model 2** | | | **Model 3** | | | **Model 4** | |
|  | Estimate | 95% CI |  | Estimate | Estimate |  | Estimate | 95% CI |  | Estimate | 95% CI |
| Age (years) | -0.77 | (-2.2, 0.7) |  | -0.32 | (-1.9, 1.27) |  | -0.24 | (-1.7, 1.23) |  | -0.30 | (-1.73, 1.13) |
| Sex (Male) | -4.47 | (-13.8, 4.9) |  | -1.1 | (-10.49, 8.28) |  | -4.58 | (-13.61, 4.46) |  | -5.75 | (-14.67, 3.17) |
| BMI (kg/m2) | 1.43^*^ | (0.7, 2.2) |  | 1.42^*^ | (0.65, 2.2) |  | 1.35^*^ | (0.63, 2.07) |  | 1.42* | (0.71, 2.12) |
| LVIDd Z-score | 7.44^*^ | (3.57, 11.32) |  |  |  |  |  |  |  | 3.75 | (-0.97, 8.47) |
| LVIDs Z-score |  |  |  | 5.13^*^ | (1.39, 8.86) |  |  |  |  |  |  |
| LV Mass Z-score |  |  |  |  |  |  | 7.83^*^ | (3.55, 12.10) |  | 5.86* | (1.03, 10.70) |

^*^p-value < 0.05.

| **Table S3.** Estimated regression coefficient with 95% confidence interval (CI) obtained from multivariable regression models for the analysis of the association between Ve/VCO_2_ and echocardiographic measures of LV size | | | | | | | | | | | |
| --- | --- | --- | --- | --- | --- | --- | --- | --- | --- | --- | --- |
|  | **Model 1** | | | **Model 2** | | | **Model 3** | | | **Model 4** | |
|  | Estimate | 95% CI |  | Estimate | 95% CI |  | Estimate | 95% CI |  | Estimate | 95% CI |
| Age (years) | -0.08 | (-0.38, 0.21) |  | -0.07 | (-0.39, 0.24) |  | -0.11 | (-0.42, 0.19) |  | -0.11 | (-0.42, 0.2) |
| Sex (Male) | -3.7^*^ | (-5.63, -1.77) |  | -3.79^*^ | (-5.72, -1.86) |  | -3.35^*^ | (-5.28, -1.41) |  | -3.37* | (-5.36, -1.38) |
| BMI (kg/m2) | -0.13 | (-0.29, 0.03) |  | -0.12 | (-0.29, 0.05) |  | -0.12 | (-0.28, 0.04) |  | -0.12 | (-0.29, 0.04) |
| LVIDd Z-score | -0.21 | (-0.98, 0.56) |  |  |  |  |  |  |  | 0.1 | (-0.90, 1.10) |
| LVIDs Z-score |  |  |  | -0.12 | (-0.87, 0.62) |  |  |  |  |  |  |
| LV Mass Z-score |  |  |  |  |  |  | -0.69 | (-1.58, 0.19) |  | -0.75 | (-1.81, 0.31) |

^*^p-value < 0.05.
